# A Cross-Sectional Study on Knowledge, Attitude, and Practices Related to Cervical Cancer Screening Among the Nursing Staff in a Tertiary Care Hospital in the Western Region of India

**DOI:** 10.7759/cureus.51566

**Published:** 2024-01-03

**Authors:** Zalak V Karena, Payal S Faldu

**Affiliations:** 1 Obstetrics and Gynecology, GMERS (Gujarat Medical Education & Research Society) Medical College, Morbi, IND

**Keywords:** pap smear, cervical cancer, knowledge attitude practice survey, visual inspection with acetic acid (via), cervical cancer prevention

## Abstract

Background

Cervical cancer is the fourth most common cancer in women globally. It is one of the leading causes of cancer deaths in females in India. Cervical cancer has a long latent precancerous period from index human papillomavirus (HPV) infection to potential cancer development, making screening one of the most effective methods of cancer prevention. Despite the national cancer prevention programme for cancer cervix, with defined guidelines for cervical cancer screening by the auxiliary nurse midwives (ANM) and nurses, cervical cancer screening is very limited in India. In this study, we aim to assess the knowledge, attitude, and practices related to cervical cancer and screening methods among the nursing staff in a tertiary care hospital attached to a medical teaching institute.

Methodology

A cross-sectional study was conducted by a semi-structured questionnaire in a tertiary care hospital in Morbi, situated in the western region of India, between November and December 2023. Female nursing staff of the hospital in the age of 20 to 60 years were included as study participants. The study was approved by the Institutional Ethical Committee.

Results

In the study, 64.9% of participants were in the age group of 20-29 years, and 52.6% were unmarried, forming a major portion of the study group being of young age. Of the participants, 70.1% identified cancer of the cervix as a major public health problem. Only 28.8% of the participants had adequate and comprehensive knowledge of cervical cancer screening. Though 92.8% of the participants knew of Pap smear as a cervical cancer screening method, only 12.4% of participants were aware of the visual inspection with acetic acid (VIA) and 2% were aware of HPV testing as a tool for cervical cancer screening. Only 5.2% of the study participants had themselves been screened for cervical cancer. Of the participants, 87.6% had never taken a Pap smear, and 95.8% of participants had never taken VIA of any woman. A total of 32.3% of participants gave the reason of not having adequate skills to perform VIA as the reason for not ever having screened the patient with VIA. A total of 6.2% of participants had been trained in cervical screening methods formally.

Conclusion

The limited knowledge of the nursing staff of cervical cancer and its screening and low self-screening prevalence among healthcare professionals highlight the need to increase awareness of cervical cancer and screening to bring the impetus to training and result-driven implementation of screening programmes for cervical cancer in India.

## Introduction

As per the GLOBOCAN 2020 report, cervical cancer is the fourth most common cancer in women globally. There were an estimated 604,000 new cases and 342,000 deaths worldwide annually due to cervical cancer. The majority of new cases and deaths (approximately 85% and 90%, respectively) occur in low- and middle-income countries (LMICs) [1]. In India, as per the estimated data for 2019, there were 45,300 deaths due to cervical cancer. The crude cervical cancer incidence per 100,000 women in 2020 in India was 18 and the cumulative risk of cervical cancer in 2020 was 2% [2].

Persistent infection of the lower genital tract by one of the 15 high-risk human papillomavirus (hrHPV) types is the primary cause of cervical cancer. The human papillomavirus (HPV) infection prevalence among healthy women aged beyond 30 years is around 11.7% worldwide. Age-specific HPV infection prevalence is highest at 28% in women less than 25 years, suggesting that the HPV infection is predominantly transmitted with sexual debut [3]. The pathogenesis of cancer cervix and knowledge of HPV epidemiology resulted in the development of vaccination and screening of precancerous lesions as two important strategies for the prevention and early detection of cervical cancer. With intervention programmes at all levels of prevention, the elimination of cervical cancer is an actual possibility. HPV vaccination was launched in 2006. At the population level, there is evidence of the reduced prevalence of hrHPV types, anogenital warts, high-grade cervical abnormalities, and cervical intraepithelial neoplasia 2 (CIN2+) caused by the vaccine types HPV among young women, suggesting the effectiveness of HPV vaccination [4]. While HPV vaccination is being targeted to prevent cervical neoplasia, screening detects cervical precancerous lesions such as cervical intraepithelial neoplasia (CIN) and adenocarcinoma in situ early, to treat them effectively to prevent invasive cancer and decrease mortality rates due to cervical cancer. Therefore, screening remains a top priority for cervical cancer prevention for the next few decades at least until HPV vaccination does not become universal. Unfortunately, most LMICs lack effective intervention programmes for cervical cancer. In developed countries, the proportion of women screened by Pap test is reported to vary between 68% and 84% [5,6]. However, fewer than one in 10 women have been screened in India for cervical cancer in the last five years. While the cervical cancer vaccine has not yet been included in the National Immunization Schedule in India, a national screening programme for cervical cancer exists [2].

In 2018, the WHO Director-General made a call for worldwide action for the elimination of cervical cancer to bring cases to a threshold of four per 100,000 women worldwide. An intervention strategy was proposed at three levels of prevention. The WHO strongly recommended the cervical cancer elimination strategy by vaccinating 90% of all girls by the age of 15 years, screening 70% of women, first by the age of 35 years and then by the age of 45 years, and treating at least 90% of all precancerous lesions detected by screening and 90% cases of invasive disease [7]. In August 2020, the World Health Assembly adopted this global strategy for cervical cancer elimination. The WHO gave a framework for monitoring the cervical cancer elimination strategy to accelerate the elimination of cervical cancer as a public health problem in May 2023. The framework highlights specific indicators related to the key domains and identifies the most important indicators at each level of prevention to help track progress and drive programmatic improvements and adjustments. Cervical cancer screening coverage, cervical pre-cancer incidence, screening test positivity rate, availability of the national cervical cancer screening programme, HPV test availability in primary health care (PHC), and referral pathway for screen-positive women (linkage to treatment) are few of the screening indicators for monitoring the screening strategy [8].

The National Cancer Control Programme and the Ministry of Health in India have come up with national guidelines for screening cervical cancer with the existing healthcare system [9-11]. In resource-limited healthcare centres, visual inspection with acetic acid (VIA) is recommended for screening, where cytology is not available. The guideline has clearly defined a referral system from primary care to secondary and tertiary care for screening and treatment. The screening technique of VIA is simple enough and the guideline suggests that VIA be performed by the nursing staff and auxiliary nurse midwife (ANM), where the doctors are scarce owing to the huge workforce of nursing staff being available. Hence, it is very necessary that the nursing staff have the necessary knowledge and attitude and they inculcate the practice of cervical screening, especially VIA and cytology, which are at present the common screening methods available widely at present in India. The current study aims to assess the same as well as their attitude towards self-screening.

## Materials and methods

**Figure 1:**
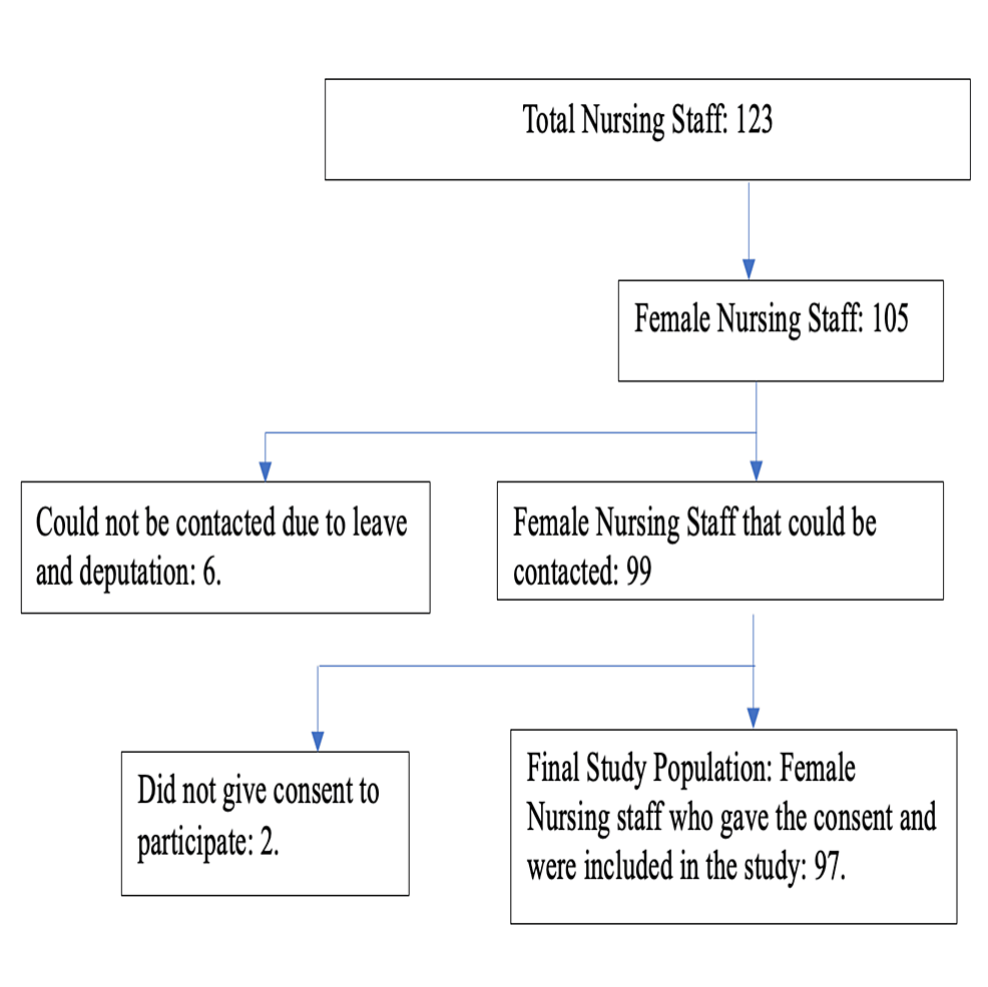
Vignette of the study participants selection

The study was a descriptive cross-sectional study carried out between November and December 2023 in a tertiary care hospital in the western region of India. The study was commenced after the approval from the Institutional Ethics Committee of GMERS Medical College, Morbi (GMERSMCM/IEC/3/2023). The study participants who could be included in the study were all the female nursing staff of the hospital from the age of 20 to 60 years, who were accessible during the study period and gave verbal consent for the survey. Of the total 105 female nurses in the hospital, six female nurses could not be included in the study due to being on leave or deputation during the survey period. Hence, a total of 97 female nursing staff were included in the study (Figure 1). The study participants were informed of the purpose of the study and the necessary information to fill out the questionnaire. After the verbal consent, they were given the link to the Google Forms (Google, Mountain View, CA) survey to fill out the semi-structured questionnaire. They filled out the form in the presence of the researcher and the forms were submitted. Incompletely filled forms, if submitted, were considered for exclusion from the study. No submission met this exclusion criterion in our study.

Demographic details of the study participants were collected. Their knowledge of cervical cancer, its precancerous state, the risk factors, symptoms, the screening methods, i.e., Pap test and VIA, periodicity of screening, eligible candidates for screening, and knowledge of the vaccine were tested. Their status of self-screening, the results of the screening and the reason, and details if not self-screened were collected. Their practice of cervical cancer screening in the hospital was collected. The details of their practice, if they manage female patients, do per-speculum examination routinely, ask the details of the screening status of the patients, if they refer patients for cervical cancer screening, and if they have ever taken Pap smear and VIA of the patients were collected and assessed as mentioned in detail in the results section. Their attitude for not having ever taken a Pap smear and VIA of the patients was analysed quantitatively through preset prompted options. The details of their formal training for VIA and Pap tests were also collected. The questionnaire was adapted from the study by Shashank Shekhar et al. with necessary modifications [12].

Statistical analysis

The data were collected through Google Forms, and compiled and analysed in a Microsoft Excel sheet (Microsoft Corporation, Redmond, WA). Scoring was done to calibrate the knowledge domain. Each question was given a score of one for complete correct knowledge and 0.5 when the participant could answer the question scarcely. A total of 12 questions were scored in the knowledge domain. A total score of more than six was considered adequate knowledge and a score equal to or less than six was considered inadequate knowledge. The representation and interpretation of the data were done in frequency and percentage proportion. The demographic details and knowledge domain of cervical cancer screening of nurses who did and did not have self-screening for cervical cancer were compared and analysed to find, if any, correlation. Statistical methods included the chi-square test using SPSS version 26 (IBM Corp., Armonk, NY). A p-value of <0.05 was considered statistically significant.

## Results

In the study group, 64.9% (63) of the participants were in the age group of 20-29 years and 52.6% (51) were unmarried, forming the major portion of the study participants being of young age and with less than five years of work experience. Of the participants, 63.5% (61) were nullipara owing to the majority being unmarried. A total of 17.5% (17) of participants had tubal ligation and 18.6% (18) used natural method of contraception. A total of 11.34% used barrier contraception and 74% of the participants who used contraception had used more than one method of contraception. No participant had a family history of cervical cancer. One participant had a family history of vulval cancer and that participant was not ever screened for cervical cancer.

Of the participants, 70.1% identified cancer of the cervix as a major public health problem. Surprisingly, 57.7% knew viral infection as a risk factor. The majority (92.8%) of participants knew that Pap smear is used for cervical cancer screening. Of the respondents, 76.3% knew that early cervical changes are easily curable. On being asked about the timing of the Pap test, only 26.8% of participants gave a correct answer. A total of 12.4% of participants were aware of the VIA as a tool for cervical cancer screening. Table 1 shows the results of the survey on knowledge. Of the participants, 68% had heard of a vaccine to prevent cervical cancer. When scores were counted for questions related to knowledge about cervical cancer and its prevention, only 28.8% of participants had adequate knowledge.

**Table 1:** Knowledge about cervical cancer and screening (n = 97) HPV: human papillomavirus; VIA: visual inspection with acetic acid.

	Knowledge about cervical cancer and screening	Frequency	Percentage
1)	Identifies cervical cancer as a public health concern	68	70.1
2)	Identifies symptoms of cervical cancer		
	Foul-smelling discharge	83	85.6
	Intermenstrual bleeding	70	72.2
	Post-coital bleeding	47	48.5
	Asymptomatic	28	28.9
	Weight loss	54	55.7
3)	Identifies risk factors of cervical cancer		
	Identifies 3 risk factors	48	49.4
	Identify more than 3 risk factors	0	0
	HPV infection	15	15.4
	Sexual intercourse	56	57.7
	Multiple sexual partners	51	52.6
	HIV	43	44.3
	Immunocompromised state	44	45.4
	Early age of first sexual intercourse	29	29.9
	Others	4	4.1
4)	Enumerates cervical cancer screening methods		
	Pap smear	90	92.8
	VIA	12	12.4
	Cervical biopsy	61	62.9
	Colposcopy	1	1
	HPV testing	2	2
5)	Identifies eligible candidates for screening		
	Symptomatic	73	75.3
	Married women	51	52.6
	*≥*30 years or after 3 years after first sexual intercourse (whichever is earlier)	51	52.6
	HIV-positive women	33	34
6)	Identifies that a Pap smear can detect pre-cancerous lesions	31	32
7)	Frequency of Pap smear screening		
	Annually	40	41.2
	Every 2 years	13	13.4
	Every 3 years	26	26.8
	Every 5 years	2	2.1
	When has symptoms	16	16.5
8)	Knowledge and understanding of VIA		
	Screening method for cervical cancer	68	70.8
	Visual test (not a laboratory test)	23	24
	Requires per speculum examination	16	16.7
	It is chemical exposure	7	7.3
9)	Identifies that VIA can detect pre-cancerous lesions	52	53.6
10)	Frequency of VIA screening		
	Annually	42	43.3
	Every 2 years	10	10.3
	Every 3 years	24	24.7
	Every 5 years	4	4.1
	When has symptoms	17	17.5
11)	Precancerous lesions are curable	74	76.3
12)	Knowledge of the availability of cervical cancer vaccine	66	68

Nearly 85.6% of participants were routinely managing female patients; however, only a few (39.2%) were doing per speculum examination when required under the circumstances of availability of the necessary instruments or being posted in the obstetrics and gynaecology department. Most of the participants (87.6%) had never taken a Pap smear, only 23.7% of participants asked patients about their cervical cancer screen status, and 85.4% of the participants had never referred patients for screening for cervical cancer. Table 2 shows the results of attitudes and practices of cervical screening among the study participants. A total of 30.6% of nurse participants believed that a Pap smear is a procedure to be done by a doctor only. The most common reason identified by the nursing staff for not doing VIA was because of not having the necessary skills to perform VIA (Table 2).

**Table 2:** Attitude and practices related to cervical cancer screening among the nursing staff VIA: visual inspection with acetic acid.

	Attitude and practices	Frequency	Percentage
1)	Self-screening		
	Self-screened	5	5.2
Attitude	Reason for getting self-screened:		
	Awareness of getting screened for cervical cancer	1	20
	Symptoms	2	40
	Other (got invitation/as part of regular body check-up)	2	40
Attitude	Reason for not getting self-screened		
	No reason	56	60.9
	Not at risk	7	7.6
	Don’t have symptoms	15	16.3
	Feeling shy or uncomfortable	8	8.7
	Afraid of the results	2	2.2
	Not proactive (if invitation given, will get done)	4	4.3
2)	Screening of the patients		
	Managing female patients	83	85.6
	Have performed per-speculum examination when required	38	39.2
	Ask history of screening for cervical cancer to the patients and other females in routine	23	23.7
	Refer patients for cervical cancer screening routinely	14	14.6
	Ever taken a Pap smear of the patient	12	12.4
Attitude	Reason for not having ever taken a Pap smear of the patient		
	Doctor’s duty	26	30.6
	Not posted in the gynaecology department	28	32.9
	Speculum not available	25	29.4
	No reason	7	8.2
	Not having the skill and knowledge to take VIA	24	28.2
	Ever taken VIA of the patient	4	4.1
Attitude	Reason for not having ever taken VIA of the patient:		
	Doctor’s duty	22	23.7
	Not posted in the gynaecology department	27	29
	Speculum not available	21	22.5
	No reason	14	15.1
	Not having the skill and knowledge to take VIA	30	32.3
	Trained for screening methods		
	Pap smear	4	4.1
	VIA	3	3

The chi-square test was used to test the association of age, parity, and knowledge with the self-screening status of study participants. Four of the five self-screened participants had scores suggesting adequate knowledge for cervical cancer (Table 3), and the association was statistically significant (p = 0.009). The age and parity did not show a statistically significant relation with self-screening.

**Table 3:** Correlation of knowledge, age, and parity to the self-screen status of the participants

		Not screened (frequency)	Screened (frequency)	P-value
Age	<30 years	63	1	0.02
	>30 years	29	4	
	Total	92	5	
Parity	Nulliparous	61	2	0.22
	Parous	31	3	
	Total	92	5	
Knowledge	Adequate (score > 6)	24	4	0.009
	Inadequate (score of 6 or less)	68	1	
	Total	92	5	

## Discussion

The majority of the nursing staff in our study had scarce knowledge of cervical cancer and the screening methods. Only half of the participants knew cervical cancer was a consequence of sexually transmitted diseases and married women were the eligible candidates for screening, which is a very poor level of knowledge to be borne by a healthcare professional. No study participant was able to enumerate more than three risk factors for cervical cancer in contrast to another similar study by Shashank Shekhar et al. (2013) in the Indian scenario [12]. Only 32% of participants in our study knew that Pap smear can detect precancerous lesions, which is less compared to the previously published study by Singh et al. (2012) [13]. The knowledge of VIA was less than Pap test, which could be alarming as VIA is a technique that is primarily targeted to be conducted by the nursing staff for cervical cancer screening in low-resource areas. In our study, 92.8% were aware of the Pap test as a screening method, compared to 68.9% in a similar study conducted in Pakistan [14]. However, awareness of VIA as a recall question was 12.4% at the start of the study, but with further succession of the study, with prior priming effect, 70.8% of participants were able to identify VIA as a screening method for cervical cancer. Very scarce knowledge on the VIA procedure stresses the need to review the curriculum of nursing staff. Also, there is a need to take large-scale actions for sensitization and formal training in cervical cancer prevention and screening amongst the nursing staff in a more effective manner. The programme of cervical cancer screening requires a grassroots penetration in the healthcare system. Half of the participants had no reason for not getting self-screened, which reflects a lack of awareness of the cancer and its consequences. Of the participants, 7.6% gave a reason of "not being at risk" for not getting self-screened, which is less than the study by Rahman et al. (2015) [15]. The association between knowledge and self-screening of the participants was found to be significant in our study, which is similar to the findings of the study by Shashank Shekhar et al. (2013) [12]. However, it needs to be highlighted that a large number of the participants who had adequate knowledge of cervical cancer were not self-screened. It implies that not necessarily knowledge brings about an attitude or change in behaviour and practice unless there is a strong impetus. Besides this, half of the participants being unmarried in our study would not be sexually active and also as sexual relations outside marriage is a social taboo in India, with that fear, the unmarried but sexually active may not get self-screened despite having the knowledge of cervical cancer screening. The limitations of our study were the small sample size, the study being quantitative in nature, and the subjective aspects of attitude and behaviour for screening could not be explored in detail.

## Conclusions

Limited knowledge of cervical cancer and screening in healthcare professionals suggests even poorer knowledge in the women in the general population. Hence, cervical cancer screening will be health care's mantle. Healthcare-initiated screening drive by devising a system of scheduled invitations for screening of all eligible females (at least so that they get screened a minimum of twice in their lifetime as recommended by the WHO) could be done, which can be successful only after creating a huge trained nursing workforce available for screening. The wider outreach of trained ANMs and nurses for cervical screening at the community level is required. Hence, large-scale training of community nursing staff for performing VIA is required to reach the target population for screening. With the increased screening by ANMs and nursing staff at the community level and with clearly defined referral pathways, tertiary care centres will cater to higher referrals from the community-screened population for re-screening and triage of suspicious cases, to perform colposcopy, biopsy, and treatment. While the doctors will remain occupied with the referral cases, the nursing staff in the tertiary care centre should be equipped with the knowledge and training to perform the basic cervical cancer screening methods of Pap smear and VIA. While the training of the community nurses will occur in a phasic manner, and meanwhile the community cervical cancer screening coverage is not yet rampant in India, preparing the nursing staff in tertiary care with necessary knowledge and skill will increase the opportunistic screening in the tertiary healthcare facilities, increase referral for screening from the general population, and increase their self-screening. The limited knowledge, poor attitude, and practices related to cervical screening in the nursing staff highlight the need for impetus in large-scale training and result-driven implementation of screening programmes for cervical cancer in India. A huge trained nursing workforce is of paramount importance in improving cervical cancer screening indicators.

## References

[1] Sung H, Ferlay J, Siegel RL, Laversanne M, Soerjomataram I, Jemal A, Bray F (2021). Global Cancer Statistics 2020: GLOBOCAN estimates of incidence and mortality worldwide for 36 cancers in 185 countries. CA Cancer J Clin.

[2] (2023). Cervical cancer India 2021 country profile. https://www.who.int/publications/m/item/cervical-cancer-ind-country-profile-2021.

[3] (2023). Human papillomavirus and related diseases in the world. Summary report. https://hpvcentre.net/statistics/reports/XWX.pdf.

[4] Drolet M, Bénard É, Pérez N, Brisson M (2019). Population-level impact and herd effects following the introduction of human papillomavirus vaccination programmes: updated systematic review and meta-analysis. Lancet.

[5] Kwon HT, Solomon FM, Nguyen S (2006). A needs assessment of barriers to cervical cancer screening in Vietnamese American health care providers. CJHP.

[6] Swan J, Breen N, Coates RJ, Rimer BK, Lee NC (2003). Progress in cancer screening practices in the United States: results from the 2000 National Health Interview Survey. Cancer.

[7] (2023). World Health Organization (WHO). WHO Director-General calls for all countries to take action to help end the suffering caused by cervical cancer. https://www.who.int/director-general/speeches/detail/cervical-cancer-an-ncd-we-can-overcome.

[8] (2023). World Health Organization (WHO). Framework for monitoring the implementation of the WHO global strategy to accelerate the elimination of cervical cancer as a public health problem. https://www.who.int/publications/m/item/framework-for-monitoring-the-implementation-of-the-who-global-strategy-to-accelerate-the-elimination-of-cervical-cancer-as-a-public-health-problem.

[9] (2023). Training manual on cervical cancer screening using visual inspection with acetic acid (VIA). https://main.icmr.nic.in/sites/default/files/Books/viamanual.pdf.

[10] (2023). Operational guidelines. Prevention, screening and control of common non-communicable diseases: hypertension, diabetes and common cancers (part of comprehensive primary health care). https://nhsrcindia.org/sites/default/files/2021-06/Operational%20Guideline%20Comprehensive%20Primary%20Health%20Care.pdf.

[11] (2023). Operational framework. Management of common cancers. https://main.mohfw.gov.in/sites/default/files/Operational%20Framework%20Management%20of%20Common%20Cancers_1.pdf.

[12] Shekhar S, Sharma C, Thakur S, Raina N (2013). Cervical cancer screening: knowledge, attitude and practices among nursing staff in a tertiary level teaching institution of rural India. Asian Pac J Cancer Prev.

[13] Singh E, Seth S, Rani V, Srivastava DK (2012). Awareness of cervical cancer screening among nursing staff in a tertiary institution of rural India. J Gynecol Oncol.

[14] Majid E, Shaikh MA, Qazi OA, Khan S, Majeed I, Bano K (2022). Awareness, screening, practices and attitudes of cervical cancer among doctors and nursing staff working at a tertiary care centre. J Pak Med Assoc.

[15] Rahman H, Kar S (2015). Knowledge, attitudes and practice toward cervical cancer screening among Sikkimese nursing staff in India. Indian J Med Paediatr Oncol.

